# A solitary presentation of panniculitis in a patient with a history of breast cancer

**DOI:** 10.1016/j.amsu.2018.10.012

**Published:** 2018-10-17

**Authors:** Irean Garcia-Hernandez, Carlos A. Lopez-Garcia, Servando Cardona - Huerta, Rocio Ortiz-Lopez, Jaime J. Tamez Salazar, Mauricio Canavati Marcos, Eduardo Esteban-Zubero, David Verdin Gonzalez, Paloma del C. Monroig-Bosque, Gabriela Sofia Gomez-Macias

**Affiliations:** a Tecnologico de Monterrey, Hospital San Jose, Servicio de Patologia, Mexico; b Tecnologico de Monterrey, Hospital San Jose, Centro de Tratamiento de Mama, Mexico; c Tecnologico de Monterrey, Escuela de Medicina y Ciencias de La Salud, Mexico; d Universidad Autonoma de Nuevo Leon, Facultad de Medicina, Mexico; e Rioja Salud, Hospital San Pedro, Departamento de Urgencias, Logroño, Spain; f Department of Pathology and Genomic Medicine, Houston Methodist Hospital, Houston, TX, United States

**Keywords:** Breast panniculitis, Autoimmune disease, Lobar septal

## Abstract

**Introduction:**

Panniculits presents as an inflammation of the subcutaneous adipose tissue of the skin. In breast, panniculitis is very rare and is usually a manifestation of underlying inflammatory conditions. The typical presentation is palpable tender nodules, which in cases of breast panniculitis, triggers an extensive work up to exclude a malignancy. Herein we present a case of septal and lobar panniculitis in a female with clinical history of invasive ductal carcinoma.

**Presentation of the case:**

A 52-year old female with past medical history of invasive breast carcinoma 5 years prior to the presentation. The patient's chief complaint was a 1-year history of a subcutaneous nodular lesion on her left breast. A core biopsy of the firm nodule showed marked inflammation of the breast. A second skin biopsy showed an abundant chronic inflammatory infiltrate, with lymphocytic vasculitis and neuritis, suggestive of an underlying autoimmune process.

**Discussion:**

Subcutaneous panniculitis with or without vasculitis is a rare condition when presenting in the breast. Panniculitis can mimic malignancy and thus, it is important to differentially diagnose it from breast carcinoma. Histologically, it is classified in lobular and septal lymphocytic panniculitis depending on specific diagnostic characteristics.

**Conclusion:**

Panniculitis of the breast is a rare condition that needs to be included in the differential diagnosis of subcutaneous breast masses. In all cases, but specifically in females with history of breast cancer, panniculitis still should be thought of as a possibility, and imaging as well as other diagnostic techniques can aid in making the correct diagnosis.

## Highlights

• Panniculitis of the Breast is a rare condition.
• Panniculitis typically is related to autoinmmune entities.
• Differential diagnosis with breast carcinoma.

## 1 Introduction

Panniculitis refers to a broad spectrum of diseases involving inflammation of the subcutaneous fat layer of the skin. It tends to present clinically in the upper trunk, but can also present in upper extremities, face etc. Improvement in diagnostic techniques has allowed identification of different entities, including lupus panniculitis, panniculitis associated with pancreatic disease, histiocytic cytophagic panniculitis, alpha-1 antitrypsin deficiency panniculitis, and others [1]. Breast panniculitis is a rare entity. The nodules formed in breast panniculitis are usually tender and may be visualized on breast imaging. Histologically, necrotic fat nodules with associated thickening and inflammation of the overlying subcutaneous fat are seen. A few cases of primary panniculitis have been reported, however, most of them have been associated with autoimmune processes [2]. Herein, we report a case of breast panniculitis exhibiting solitary lesions.

## 2 Presentation of the case

The patient was a 52-year-old Mexican postmenopausal woman, with past medical history of invasive ductal carcinoma, status-post neo-adjuvant chemotherapy, lumpectomy (with axillary lymph node dissection) and radiotherapy, 5 years prior to current presentation. In addition, the patient had autoimmune hypothyroidism, vitiligo, and had been recently diagnosed type-2 diabetes mellitus. Her relevant family history included several autoimmune disease including hypothyroidism, rheumatoid arthritis, and Addison's disease.

She presented to our institution with a 1-year history of a red, raised skin lesion on her left breast. On physical examination, an irregular, erythematous violaceous plaque of 6 cm in diameter, was identified in the superior internal quadrant of her left breast. Upon palpation, the lesion was indurated, edematous, and had an irregularly roughened texture. No additional lesions were identified elsewhere in the body.

**Fig. 1:**
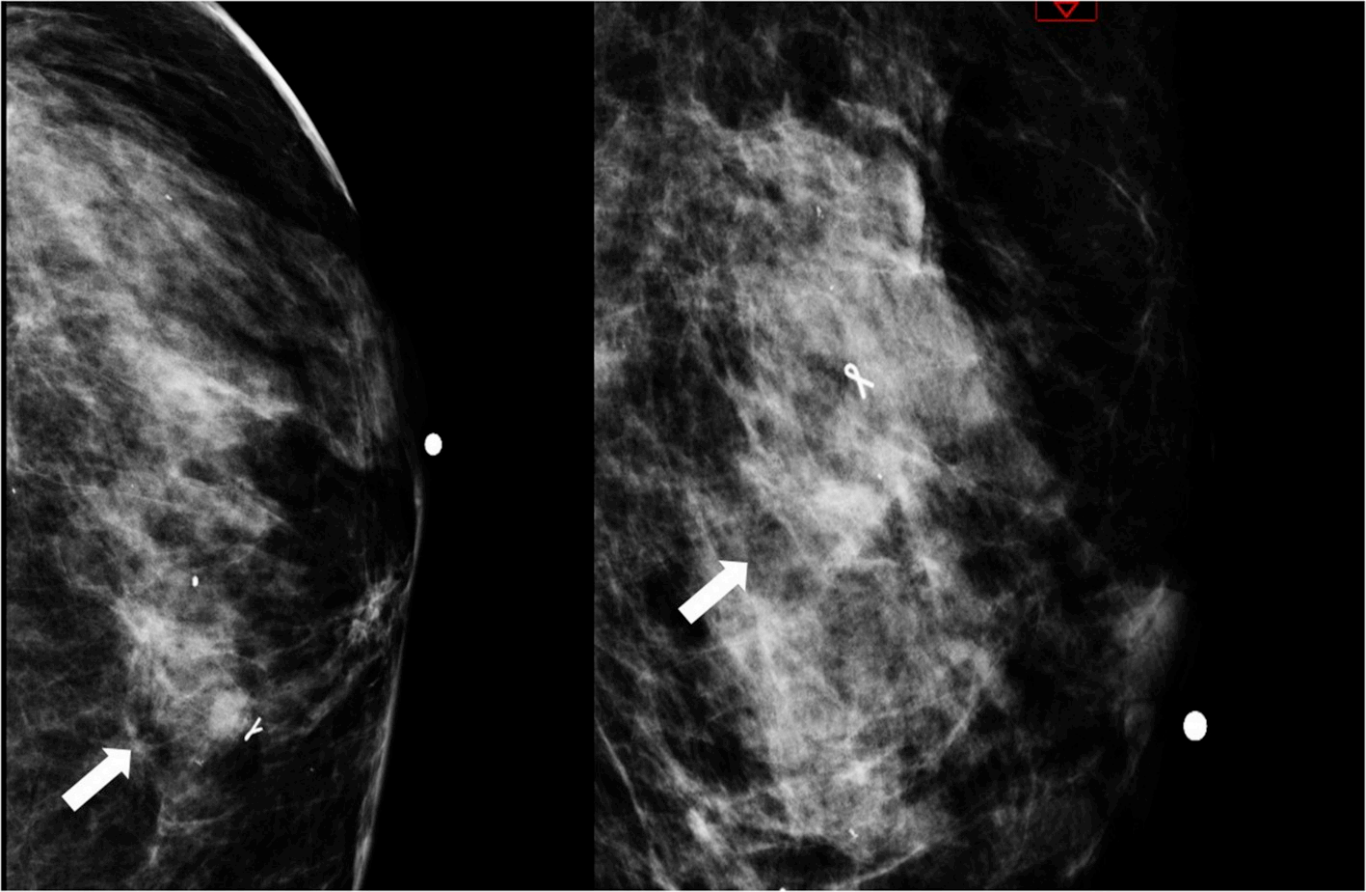
Thickening of the skin associated with cellular tissue edema; BIRADS 4b (white arrow in both pictures).

**Fig. 2:**
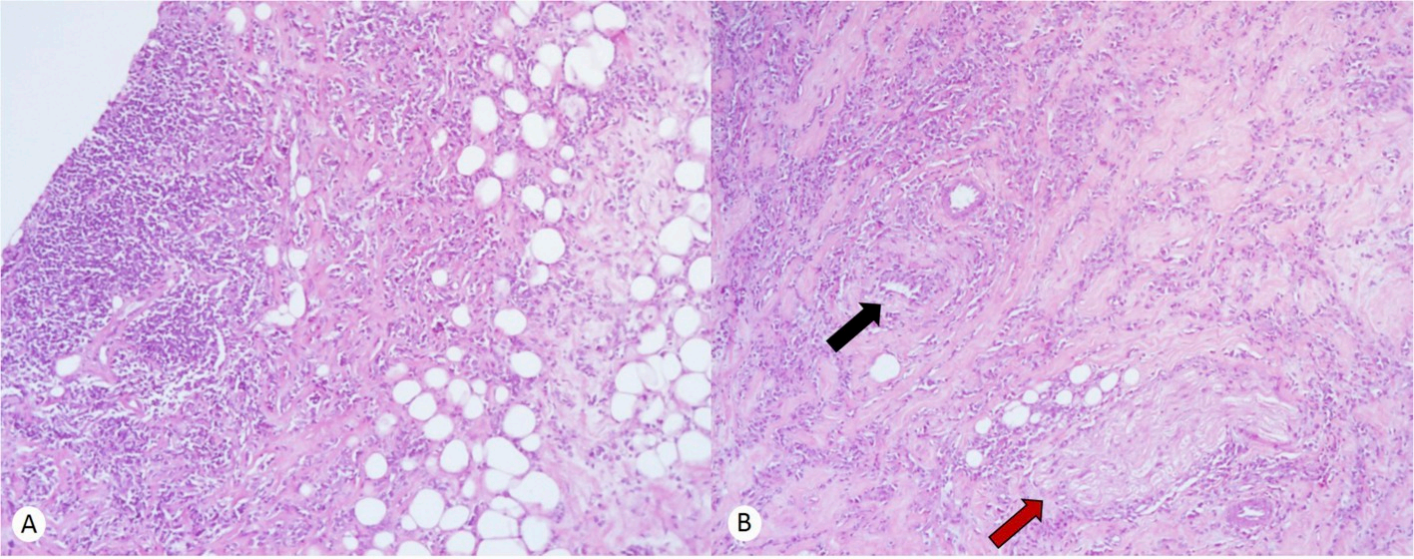
(A) Lobar and septal panniculitis, (B) Panniculitis associated with vasculitis and neuritis (black and red arrow, respectively). (For interpretation of the references to color in this figure legend, the reader is referred to the Web version of this article.)

A mammogram was performed and showed a focal thickened area over the skin, associated with subcutaneous edema, and a superficial nodule classified as BI-RAS 4b. Given the findings, a subsquent biopsy was performed (Fig. 1). Microscopic examination of the core biopsy showed clusters of small-to medium-sized inflammatory cells infiltrating collagenous stroma and adjacent adipose tissue. The infiltrate consisted of some lymphocytes, nonetheless, the majority of the cells were sheets and single filing plasma cells. Different sections showed perivascular and perineural patterns of infiltration. No giant cells or granulomas were identified (Fig. 2). Furthermore, no mammary gland tissue was seen. Immunohistochemical stains, including ER, PR, HER2-Neu, Cytokeratin, and E-cadherin, were performed; they were all negative, thus excluding the possibility of an epithelial neoplasm, specially a recurrence of her breast carcinoma.

**Fig. 3:**
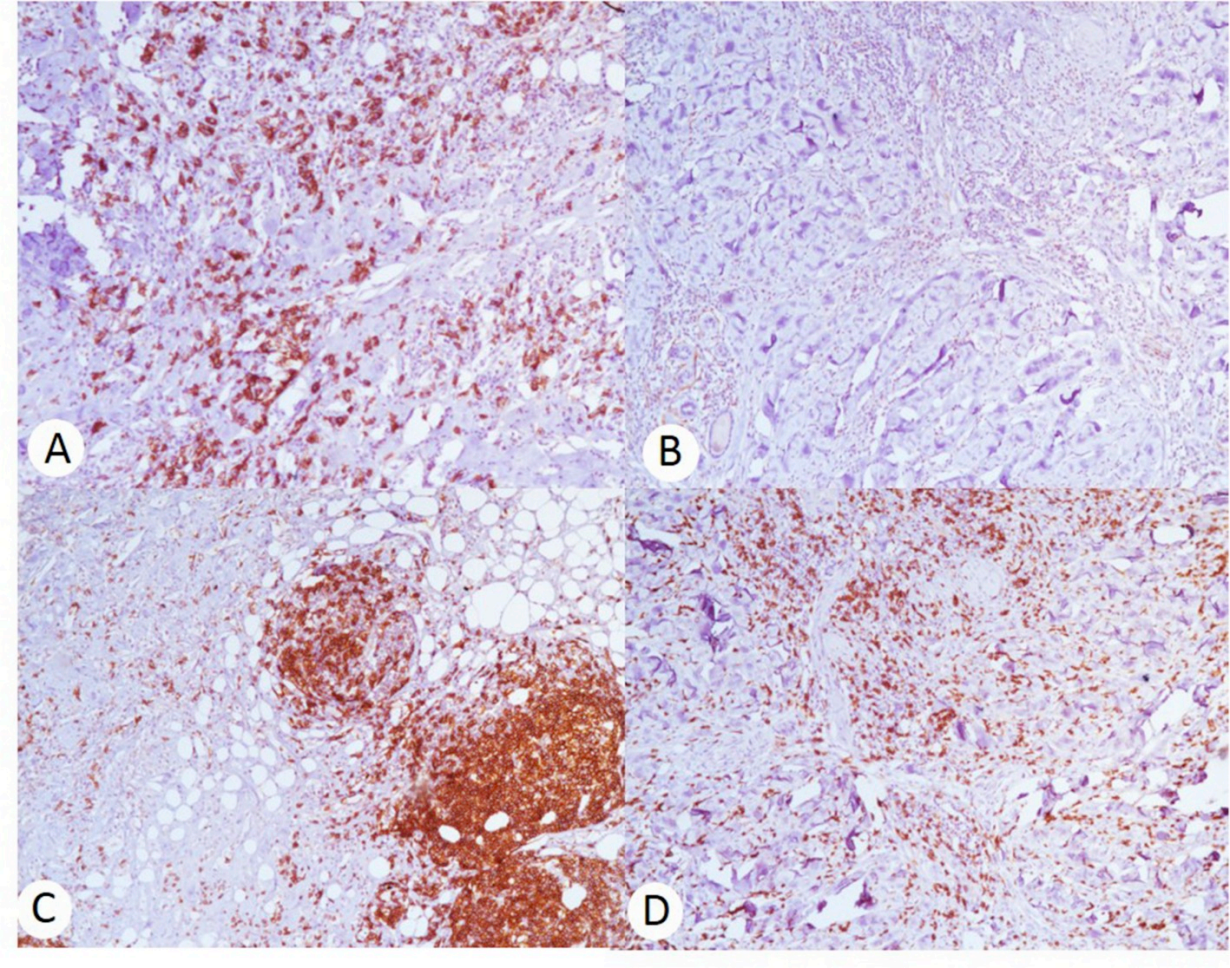
Immunohistochemistry (A) CD138-positive in plasmatic cells, (B) CD56-negative, (C) CD20-positive in reactive lymphoid follicles, and (D) CD8-positive in T-lymphochytes.

Aside from the biopsy, a fragment of skin with subcutaneous tissue was collected. On gross examination, several abnormal erythematous areas were seen. The histological sections showed subcutaneous tissue with abundant chronic inflammatory infiltrate; showing primarily plasma cells. Additionally, some lymphoid follicles and histiocytes were seen. Lymphocytic vasculitis and neuritis were also confirmed. Immunohistochemical staining was performed for: ALK, S100, desmin, and CD34. All the aforementioned stains were negative, thereby excluding an inflammatory pseudotumor. A panniculitis-type lymphoma was also considered; for this reason, T-lymphocyte (CD3, CD4, CD8) and B-lymphocyte (CD20) markers were tested by immunohistochemistry (Fig. 3). The stainings showed a mixed population of T and B cells was seen, in a ratio of 80/20. More so, CD138 was strongly positive in plasma cells. Altogether, the histological features confirmed the presence of lobular- and septal-type panniculitis. More so, the suggestion of an autoimmune mediated role in the lesion was suspected.

## 3 Discussion

Subcutaneous panniculitis is related to a broad spectrum of nonsuppurative inflammatory processes involving the subcutaneous tissue. It can also be related to vasculitis, infection or malignant neoplasia. It is difficult to diagnose, both clinically and histologically [1]. Secondary panniculitis can affect people of all ages and is more prevalent in females [2]. Clinically, it presents tender, raised, red-colored, warm nodules, frequently localized in the lower extremities and trunk. Red nodules that develop into erythematous-violaceous plaques are usually observed within a few days [3]. Histologically, the lesion has an inflammatory infiltrate, blood vessels, and fibrous septa. Panniculits could be lobar or septal with or without vascular involvement, and is classified as septal panniculitis and mixed lobar panniculitis with or without vasculitis [2,3]. It is considered to be a non-neoplastic, benign process. It is theorized to be mediated by T cells [4].

Breast panniculitis is a rare disease, especially when presenting as solitary mass. Upon physical examination, the presence of a mass makes the consideration of a breast carcinoma highly likely. However, excluding this benign, non-neoplastic diagnosis is very important [5]. As mentioned above, panniculitits can have various etiologic factors. It is an inflammatory, non-suppurative processes involving subcutaneous fat, which is the reason why patients usually suffer from constitutional symptoms as well [6].

The radiological findings of subcutaneous panniculitis with or without vasculitis may vary depending on the stage of the disease [6]. The lesions are usually seen as hyperechoic zones within hypoechoic zones within the subcutaneous tissue with increased echogenicity in the ultrasound [7]. Half of the cases of breast panniculitis present as components of a systemic disease [8]. In our case, the histological findings were compatible with lobar and septal panniculitis that involved small blood vessels.

Lobar panniculitis can be associated with idiopathic nodules, trauma, sickle cell disease, alpha-1 antitrypsin deficiency, pancreatic disease, systemic lupus erythema, scleroderma, rheumatoid arthritis, erythema induratum, and paraneoplastic disease. Septal panniculitis may be associated with erythema nodosum, eosinophilic fasciitis, and polyarteritis nodosa. Finally, mixed panniculitis may occur in lupus profundus and subcutaneous sarcoidosis. If vasculitis is associated with panniculitis, then polyarthritis nodosa, erythema nodosum, and nodular vasculitis should be considered [8].

In our case, the diagnosis was compatible with mixed panniculitis associated with vasculitis and neuritis. The disease was limited to the breast at the time of diagnosis and follow-up. This condition can present as a solitary lesion or as part of a systemic disease [9]. Breast panniculitis has rarely been described and it is important to take it into consideration in differential diagnosis of subcutaneous breast masses. A thorough history and clinical work up might be needed to exclude malignancies and systemic diseases. Very few cases of breast panniculitis associated with vasculitis have been reported in the literature; however, all cases have had an excellent prognosis with surgical resection and corticosteroid treatment. In the case of our patient, the disease manifested localized in the breast (and not visceral organs), thus, the patient had a good overall prognosis.

## 4 Conclusion

Panniculitis of the breast is a disease mostly associated with systemic symptoms. Due to this, it can be challenging to make the correct diagnosis. Breast panniculitis should be considered in all cases of patients presenting with an isolated subcutaneous mass or nodule. Even in cases with breast cancer (where the main suspicion is disease recurrence) the presentation of an inflammatory, benign, non-neoplastic lesion merits consideration. The clinical presentation, along with the findings on imaging and tissue sampling can altogether contribute to the diagnosis. This case report has been described in accordance with the SCARE criteria [10].

## Provenance and peer review

Not commissioned, externally peer reviewed.

## Ethical approval

Our study is exempt from ethnical approval.

## Sources of funding

I don't have any sources in my research.

## Author contribution

Garcia Hernandez Irean: Study concepts, study design, manuscript editing and review.

Lopez Garcia Carlos A.: Study concepts, study design, manuscript editing and review.

Cardona Huerta Servando: Data acquisition, manuscript editing manuscript review.

Ortiz Lopez Rocio: Data acquisition, manuscript editing manuscript review.

Tamez Salazar Jaime J.: Data acquisition, manuscript editing manuscript review.

Canavati Marcos Mauricio: Data acquisition, manuscript editing manuscript review.

Verdin Gonzalez David: Data acquisition, manuscript editing, manuscript review.

Eduardo Esteban Zubero: Manuscript review, manuscript editing.

Paloma del C. Monroig-Bosque: manuscript editing, manuscript review.

Gomez Macias Gabriela Sofia: Data acquisition, manuscript editing manuscript review.

## Conflicts of interest

We don't have any conflicts of interest.

## Research registration number

This study does not require declaration.

## Guarantor

I Gabriela Sofia Gomez Macias accept the full responsibility for the work and conduct of the study.

## Consent

Written informed consent was obtained from the patient for publication of this case report and accompanying images. A copy of the written consent is available for review by the Editor-in-Chief of this journal on request.

## Appendix A Supplementary data

SCARE Guideline Checklist SCARE Guideline Checklist

SCARE Guideline Checklist PAN SCARE Guideline Checklist PAN
